# Effect of an Eight-Week Breathing Exercise Program on the Respiratory Function and Craniovertebral Angle in Dental Students: A Pre-test and Post-test Quasi-experimental Study

**DOI:** 10.7759/cureus.101118

**Published:** 2026-01-08

**Authors:** Elshaimaa Hafez, Ethar Ashraf, Shaikha Saeed, Maryam Essam, Veena Raigangar, Hatem Ahmed

**Affiliations:** 1 Physical Therapy, University of Sharjah, Sharjah, ARE; 2 Internal Medicine, Tower Health Medical Group, Phoenixville, USA

**Keywords:** breathing exercises, craniovertebral angle, dental students, forced vital capacity (fvc), forward head posture, neck posture, peak expiratory flow, postural correction, respiratory function tests, spirometry

## Abstract

Introduction

Dental training requires prolonged static and forward-leaning working positions that may contribute to forward head posture. Forward head posture can be quantified using the craniovertebral angle (CVA) and may be associated with reduced respiratory performance. This study investigated whether an eight-week breathing exercise program improves respiratory function and neck posture in senior dental students.

Methods

A prospective pre-test/post-test quasi-experimental study was conducted among senior dental students (20-25 years) at the University of Sharjah. Forty-five participants completed baseline testing; 40 completed post-intervention testing and were included in paired analyses. Respiratory function was assessed using spirometry (forced vital capacity (FVC), forced expiratory volume in 1 second (FEV₁), peak expiratory flow (PEF)). Neck posture was assessed using CVA from standardized lateral photographs. The intervention consisted of daily diaphragmatic breathing, alternate-nostril breathing, and dynamic breathing exercises for eight weeks. Pre-post changes were analyzed using paired-sample t-tests.

Results

Respiratory function improved significantly after the intervention. Mean FVC increased from 2.99 ± 0.90 L to 3.32 ± 0.87 L (p < 0.001), mean FEV₁ increased from 2.81 ± 0.89 L to 3.17 ± 0.84 L (p < 0.001), and mean PEF increased from 6.50 ± 2.31 L/s to 7.42 ± 2.08 L/s (p < 0.001). CVA did not change significantly (42.95 ± 8.11° pre-intervention vs 43.35 ± 8.23° post-intervention; p = 0.83). Mean spirometry values remained below device-generated predicted/reference values despite improvement.

Conclusion

An eight-week breathing exercise program significantly improved spirometric measures in dental students but did not significantly change CVA. These findings suggest that breathing exercises can enhance respiratory function, while meaningful correction of forward head posture may require additional posture-specific interventions.

## Introduction

Posture is a key ergonomic factor because neutral joint alignment reduces mechanical strain on the musculoskeletal system, whereas sustained or non-neutral postures increase biomechanical loading and are recognized risk factors for musculoskeletal symptoms [1]. Dental professionals are particularly vulnerable because clinical work commonly involves prolonged static neck/trunk positions, which have been consistently linked with high rates of musculoskeletal complaints in this occupation [2]. Accordingly, the prevalence of musculoskeletal pain among dental practitioners frequently exceeds 60%, most commonly affecting the neck, back, and shoulders [2-5].

Posture also has important implications for respiratory function. Efficient breathing depends on thoracic expansion and coordinated diaphragmatic movement; however, forward head posture (FHP) can disrupt thoracic biomechanics and limit rib cage mobility, thereby reducing lung expansion [6]. FHP may also impair diaphragmatic efficiency and increase reliance on accessory respiratory muscles, contributing to reduced pulmonary performance [7]. Consistent with these mechanisms, reduced forced vital capacity (FVC), forced expiratory volume in one second (FEV₁), and peak expiratory flow (PEF) have been reported in individuals with FHP compared with those with neutral head posture [8].

Breathing exercises may enhance respiratory performance by improving breathing mechanics and respiratory muscle function. Diaphragmatic breathing has been associated with increased chest expansion, reduced respiratory rate, and improved oxygenation efficiency [9]. Controlled breathing techniques, including alternate-nostril breathing, may also improve airflow regulation [10]. However, despite the potential value of these approaches, evidence specific to dental populations remains limited compared with broader adult cohorts.

Given that postural deviations may contribute to reduced lung capacity [7,8], breathing exercises could represent a practical preventive strategy for dental professionals. Therefore, this study aimed to investigate the effect of an eight-week breathing exercise program on respiratory function and neck posture in dental students.

## Materials and methods

Study design, setting, and timeline

This study used a pre-test/post-test, prospective, quasi-experimental design to evaluate changes in respiratory function and neck posture following an eight-week breathing exercise intervention in dental students. The study was conducted at the College of Dental Medicine, University of Sharjah, Sharjah, United Arab Emirates. Recruitment took place in January 2017; baseline testing and the intervention period occurred between 1 March and 1 June 2017, baseline assessments were completed within 14 days of enrollment, and post-intervention assessments were performed within 14 days of completing the eight-week program.

Participants and sampling

Senior undergraduate dental students were invited to participate via in-class announcements, email notices, and clinic briefings. Convenience sampling was used, enrolling the first eligible students who consented. Eligibility was confirmed using a structured screening form (demographics, clinical training exposure, health history, and exclusion criteria), followed by a brief screening interview conducted by a physiotherapist.

Inclusion and exclusion criteria

Participants were eligible if they were senior-level undergraduate dental students aged 20-25 years, with a BMI <30 kg/m², and engaged in regular clinical training (≥5 hours/day, ≥5 days/week, for at least 12 months). The BMI threshold was selected to reduce confounding because obesity is associated with reduced lung volumes/capacities and altered spirometric values [11].

Participants were excluded if they reported or screened positive for any of the following: a prior clinician diagnosis of a spinal structural abnormality (e.g., scoliosis, kyphosis, spondylolisthesis, vertebral fracture, other spinal deformity) or clinically significant spine-related symptoms (persistent spinal pain >3 months, pain ≥4/10 at rest or with routine activity, or prior spinal surgery); chronic neck or back pain (>3 months) requiring ongoing treatment or regular analgesic use; obesity (BMI ≥30 kg/m²); long-term medication use likely to affect cardiopulmonary function or exercise tolerance (e.g., chronic bronchodilators or inhaled/systemic corticosteroids for respiratory disease, beta-blockers, antiarrhythmics, long-term opioid therapy, sedatives such as benzodiazepines); history of malignancy involving bone or prior thoracic malignancy; neurological disorders affecting posture, breathing, or motor control (e.g., stroke, multiple sclerosis, Parkinson’s disease, epilepsy requiring ongoing medication, peripheral neuropathy, neuromuscular disease); known respiratory disease (e.g., asthma, chronic obstructive pulmonary disease (COPD)) or an acute respiratory infection within the previous two to four weeks; prior thoracic or abdominal surgery that could affect respiratory mechanics; current smoking or smoking cessation within the previous five years; current pregnancy or within six months postpartum; chronic systemic inflammatory/autoimmune disease (e.g., systemic lupus erythematosus, rheumatoid arthritis, ankylosing spondylitis/axial spondyloarthritis); or active cardiac disease (e.g., heart failure, myocardial infarction within six months, uncontrolled arrhythmia).

Eligibility screening was based on participant self-report (health history questionnaire) and a standardized physiotherapist interview; no imaging or laboratory testing was performed.

Ethical considerations

Ethical approval was obtained from the Research Ethics Committee, College of Health Sciences, University of Sharjah (Approval No. REC-17-03-04-01-S). Written informed consent was obtained in Arabic and English. Participants were informed of voluntary participation, confidentiality, and their right to withdraw at any time without penalty. If withdrawal occurred, data from that participant were excluded from analysis.

Intervention

Participants completed a daily breathing program for eight weeks that included diaphragmatic breathing (nasal inhalation emphasizing abdominal expansion with minimal upper-chest motion, followed by slow pursed-lip exhalation); alternate-nostril breathing (alternating inhalation and exhalation through the left and right nostrils); and dynamic breathing exercises comprising the windmill (coordinated arm elevation overhead synchronized with inhalation and exhalation to promote thoracic mobility) and seated trunk rotations (upper-trunk rotation synchronized with breathing, exhaling into rotation and inhaling while returning to neutral).

Participants received standardized in-person instruction and demonstration by a physiotherapist at baseline and practiced each technique under supervision to confirm understanding. The daily prescription was three sets of five repetitions per technique, with a 10-second rest between sets. After the initial supervised session, exercises were performed independently. Adherence was supported by daily reminders via phone/text and a self-report checklist/log. A typical daily session required approximately 10-15 minutes, depending on the participant’s breathing pace.

**Figure 1:**
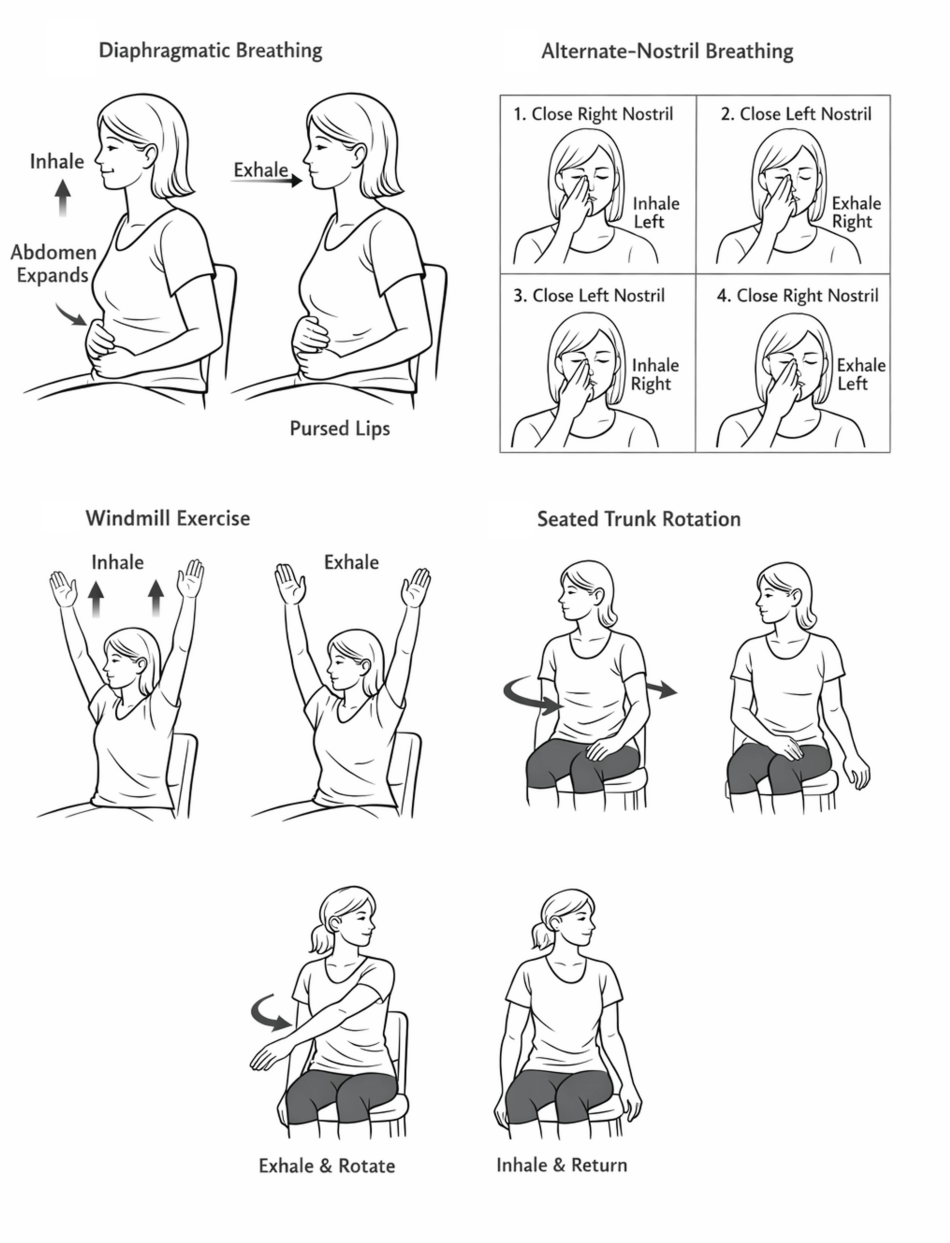
Illustrated instructions for the breathing exercise intervention, including diaphragmatic breathing, alternate-nostril breathing, windmill breathing, and seated trunk-rotation breathing

Standardized written and visual instructions were provided to all participants for each breathing technique. The diaphragmatic breathing, alternate-nostril breathing, windmill, and seated trunk rotation exercises are illustrated in Figure 1.

Outcomes and data collection procedures

All assessments were conducted by a physiotherapist trained in spirometry and postural assessment, using standardized instructions at both pre-test and post-test.

Spirometry was performed using a portable spirometer (Spirolab II-MIR, Rome, Italy) to measure FVC, FEV₁, and PEF. Participants were seated in a standardized simulated dentist working posture: seated upright on a stool with hips and knees approximately 90°, feet flat on the floor, trunk neutral, shoulders relaxed, and head in a comfortable neutral position. A nose clip was applied, and a disposable mouthpiece was used for each participant to minimize cross-contamination.

Participants were coached to perform a maximal forced expiratory maneuver after full inspiration. A minimum of three acceptable maneuvers was obtained at each time point. Additional maneuvers were performed if acceptability/repeatability criteria were not met, including evidence of cough in the first second, leak or obstructed mouthpiece, submaximal effort/early termination, delayed start, or poor reproducibility (defined as the two highest FVC and FEV₁ values differing by >150 mL) [12]. Testing continued until at least three acceptable maneuvers were obtained (maximum eight attempts). The reported FVC and FEV₁ were the largest values from acceptable maneuvers, and PEF was taken as the highest acceptable value.

FHP was quantified using the craniovertebral angle (CVA) measured from standardized lateral photographs. Photographs were taken in a designated assessment area within the college using a digital camera mounted on a tripod, leveled to the horizontal plane. Participants stood barefoot in their natural, relaxed posture with arms at their sides and eyes facing forward. The tragus of the ear was marked with a skin-safe marker. The spinous process of C7 was identified clinically by palpation and confirmed by prominence during neck flexion/extension and then marked externally. A lateral photograph was taken with the camera positioned perpendicular to the sagittal plane at a specified distance of 2 m and approximately at shoulder height. CVA was calculated as the angle between a line from the tragus to C7 and a horizontal reference line [13]. A smaller CVA indicates a more pronounced FHP. CVA was analyzed as a continuous variable.

Statistical analysis

Data were analyzed using Statistical Product and Service Solutions (SPSS, version 22.0; IBM SPSS Statistics for Windows, Armonk, NY). Pre-test and post-test differences in FVC, FEV₁, PEF, and CVA were analyzed using paired-sample t-tests. Statistical significance was set at p ≤ 0.05.

## Results

Participant flow and baseline characteristics

**Figure 2:**
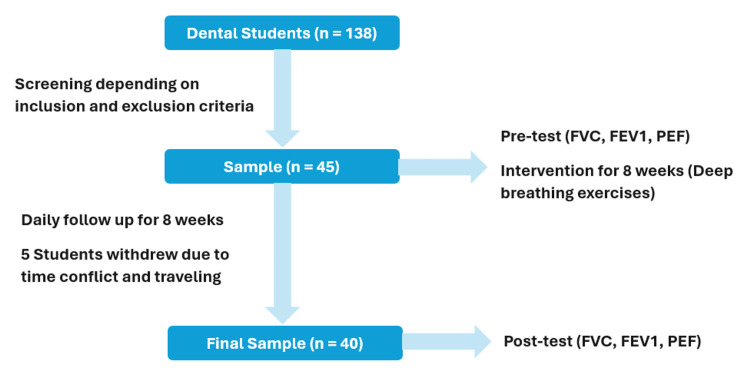
Methodology flowchart

A total of 45 dental students consented and completed baseline testing. Five participants did not complete post-intervention testing and were excluded from the paired pre-post analysis. Therefore, 40 participants (28 females, 12 males) completed both baseline and post-intervention assessments and were included in the final analysis (Figure 2). Baseline characteristics are shown in Table 1.

**Table 1:** Baseline characteristics of the participants (N = 40) Abbreviations: BMI, body mass index; SD, standard deviation

Characteristic	Total (N = 40)	Female (n = 28)	Male (n = 12)
Sex, n (%)	—	28 (70)	12 (30)
Age (years), mean ± SD	22.28 ± 0.96	22.40 ± 0.92	22.00 ± 1.04
BMI (kg/m²), mean ± SD	23.67 ± 3.28	22.50 ± 2.93	26.40 ± 2.35
Height (cm), mean ± SD	173.1 ± 8.4	170.5 ± 7.2	179 ± 8.3
Clinical practice hours (hours/day), mean ± SD	7.00 ± 0.99	7.00 ± 1.00	7.00 ± 1.00

Changes in respiratory function and neck posture

**Figure 3:**
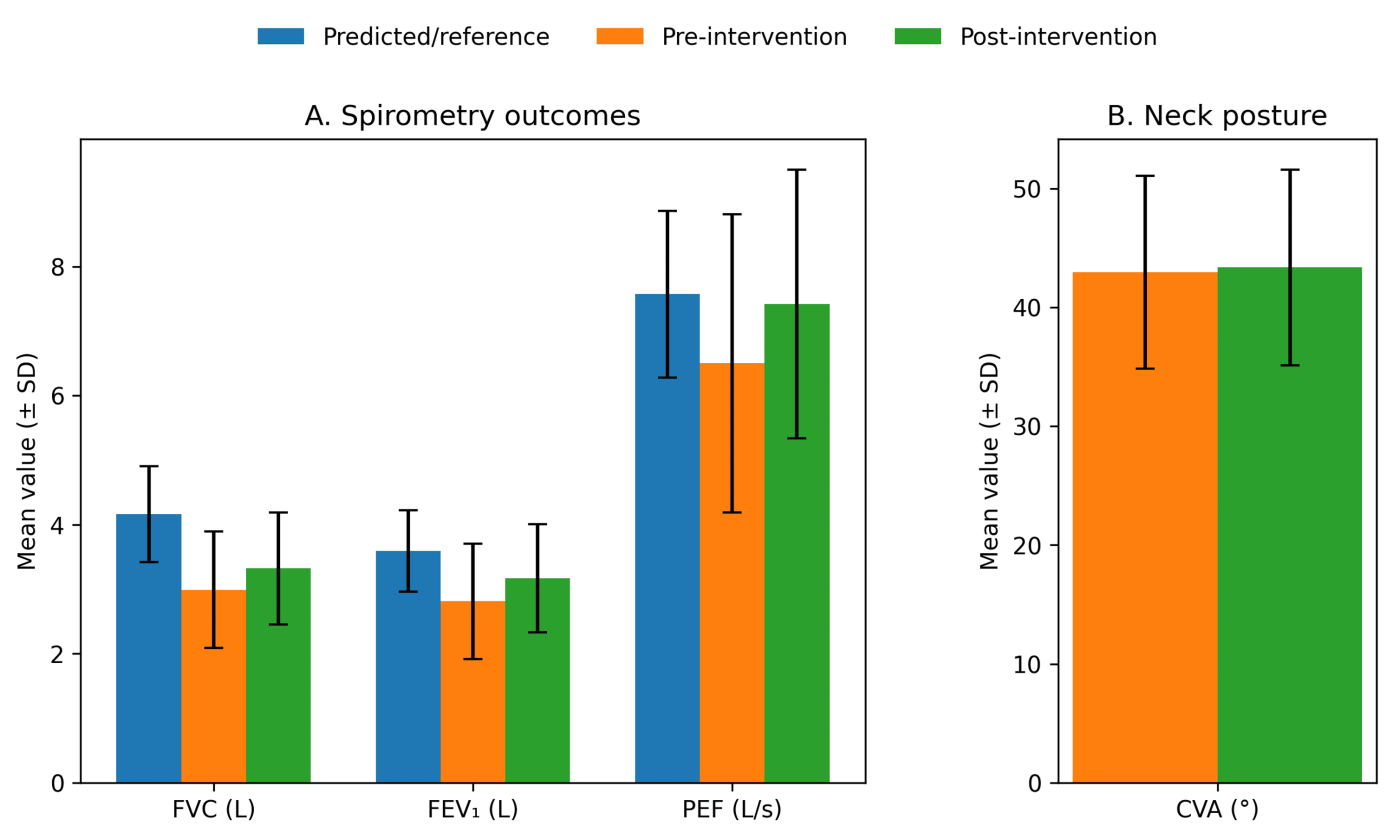
Respiratory parameters and neck posture compared with predicted/reference values Grouped bars show mean ± SD for predicted/reference values and observed pre- and post-intervention values for spirometry outcomes (Panel A: FVC, FEV₁, PEF) and craniovertebral angle (Panel B: CVA).

After the eight-week breathing exercise program, respiratory function improved significantly across all measured parameters (Table 2, Figure 3). Mean FVC increased from 2.99 ± 0.90 L to 3.32 ± 0.87 L (p < 0.001), mean FEV₁ increased from 2.81 ± 0.89 L to 3.17 ± 0.84 L (p < 0.001), and mean PEF increased from 6.50 ± 2.31 L/s to 7.42 ± 2.08 L/s (p < 0.001). These values were below the corresponding predicted/reference values at baseline and remained below predicted/reference values after the intervention (Table 2, Figure 3). Neck posture, quantified by CVA, did not change significantly from baseline to post-intervention (42.95 ± 8.11° vs 43.35 ± 8.23°; p = 0.83).

**Table 2:** Predicted/reference values and observed respiratory function and craniovertebral angle (CVA) before and after the eight-week intervention (N = 40) Predicted/reference spirometry values were obtained from the spirometer’s built-in reference equations using participant demographic inputs (e.g., age, sex, height). Abbreviations: FVC, forced vital capacity; FEV₁, forced expiratory volume in 1 second; PEF, peak expiratory flow; CVA, craniovertebral angle; SD, standard deviation

Outcome	Predicted/Reference (Mean ± SD)	Pre-intervention (Mean ± SD)	Post-intervention (Mean ± SD)	p-value
FVC (L)	4.16 ± 0.74	2.99 ± 0.90	3.32 ± 0.87	< 0.001
FEV₁ (L)	3.59 ± 0.63	2.81 ± 0.89	3.17 ± 0.84	< 0.001
PEF (L/s)	7.57 ± 1.29	6.50 ± 2.31	7.42 ± 2.08	< 0.001
CVA (°)	—	42.95 ± 8.11	43.35 ± 8.23	0.83

## Discussion

This study evaluated whether an eight-week breathing exercise program improves respiratory function (FVC, FEV₁, PEF) and neck posture (CVA) in senior dental students. Respiratory parameters increased significantly after the intervention, whereas CVA did not change significantly. Overall, the findings suggest that breathing exercises may enhance pulmonary function, but may be insufficient on their own to modify forward head posture-related neck alignment.

All spirometry outcomes improved significantly after the eight-week program, with the largest relative change seen in PEF (Table 2). One possible explanation is that breathing exercises encourage more effective use of the diaphragm, improve breathing coordination, and reduce overreliance on accessory muscles, which can support stronger and more efficient ventilation [9]. Similar improvements in respiratory measures have been reported after structured deep or controlled breathing programs in healthy young adults [10,14-15].

The marked increase in PEF is also reasonable from a testing perspective. PEF depends heavily on producing a fast, forceful exhalation immediately after a full inhalation; with practice and clearer coaching, participants often perform this maneuver more effectively [12]. For this reason, spirometry guidelines recommend repeated trials with acceptability and repeatability checks to ensure reliable results, along with consistent coaching [12]. In the present study, the same standardized spirometry instructions and quality criteria were applied at both baseline and follow-up to reduce measurement variability.

In this cohort, baseline mean spirometry values (FVC, FEV₁, PEF) were lower than the device-generated predicted/reference values and remained below predicted/reference values post-intervention despite significant improvement (Table 2, Figure 3). Predicted spirometric values depend strongly on the reference equations and population fit, and a mismatch can occur when reference equations are derived from populations that differ in anthropometrics and ethnic background from the tested cohort [16]. Therefore, the observed gap between measured and predicted values should be interpreted cautiously and not attributed to posture alone. That said, because forward head posture and altered thoracic configuration can reduce thoracic mobility and ventilatory mechanics, posture-related biomechanics remain a plausible contributing factor - particularly in a group exposed to prolonged static clinical working positions [6].

Despite improved spirometric values, CVA did not change significantly (Table 2). This suggests that breathing exercises alone may not meaningfully modify forward head posture over an eight-week period, especially when the intervention does not directly target cervical/thoracic extensor endurance, scapular stabilizer strength, or ergonomic retraining. A systematic review and meta-analysis indicates that therapeutic/postural exercise programs (often including strengthening, motor control, and stretching approaches) can produce meaningful improvements in forward head posture measures such as CVA, supporting the idea that posture correction typically requires posture-specific training rather than breathing exercises alone [17]. In addition, forward head posture classification thresholds vary across the literature (e.g., CVA <50° or ≤53° used in some studies), and CVA is commonly recommended as a continuous variable for analysis because cut-points are not universally standardized [18,19].

A plausible interpretation is that respiratory mechanics can improve through neuromuscular and behavioral adaptations in breathing pattern, even when static neck posture remains unchanged. In other words, improved ventilatory performance does not necessarily require measurable changes in CVA, particularly if thoracic expansion and breathing control improve without altering habitual head/neck alignment.

Clinical implications

For dental students who are exposed to sustained static positions - breathing exercises may represent a low-cost strategy to enhance respiratory function. However, given the absence of change in CVA, incorporating ergonomic training and targeted postural correction may be necessary to address forward head posture. Future interventions that combine breathing exercises with postural correction strategies may be more likely to influence both respiratory function and neck posture outcomes.

Limitations

Several limitations should be considered when interpreting these findings. First, the quasi-experimental pre-post design without a control group limits causal inference; observed improvements may partly reflect familiarity with spirometry maneuvers rather than the intervention alone. Second, although adherence was encouraged using reminders and self-report logs, performance of the breathing exercises was largely unsupervised after baseline training and was not objectively verified; therefore, variability in technique and true adherence could have influenced outcomes. Third, posture was assessed only using craniovertebral angle (CVA), which captures a specific aspect of sagittal head/neck alignment and does not fully characterize whole-body posture, which may also influence respiratory mechanics. Fourth, the sample consisted of a relatively small number of young dental students from a single institution, which may limit generalizability to older clinicians or broader populations. Finally, predicted/reference spirometry values were generated using the spirometer’s built-in reference equations; because predicted values depend on the chosen reference population and anthropometric/ethnic match, differences between measured and predicted values should be interpreted cautiously.

## Conclusions

An eight-week breathing exercise program significantly improved spirometric measures (FVC, FEV₁, PEF) in dental students but did not significantly change CVA. These findings suggest that breathing exercises can enhance respiratory function, while meaningful postural correction may require additional posture-specific interventions. Future controlled trials should test combined breathing and postural correction programs, include objective adherence measures, and consider reference equation suitability for the study population.

## References

[1] Soares CO, Pereira BF, Pereira Gomes MV, Marcondes LP, de Campos Gomes F, de Melo-Neto JS (2019). Preventive factors against work-related musculoskeletal disorders: narrative review. Rev Bras Med Trab.

[2] Lietz J, Kozak A, Nienhaus A (2018). Prevalence and occupational risk factors of musculoskeletal diseases and pain among dental professionals in Western countries: a systematic literature review and meta-analysis. PLoS One.

[3] Soo SY, Ang WS, Chong CH, Tew IM, Yahya NA (2023). Occupational ergonomics and related musculoskeletal disorders among dentists: a systematic review. Work.

[4] Chenna D, Pentapati KC, Kumar M, Madi M, Siddiq H (2022). Prevalence of musculoskeletal disorders among dental healthcare providers: a systematic review and meta-analysis. F1000Res.

[5] Hayes M, Cockrell D, Smith DR (2009). A systematic review of musculoskeletal disorders among dental professionals. Int J Dent Hyg.

[6] Koseki T, Kakizaki F, Hayashi S, Nishida N, Itoh M (2019). Effect of forward head posture on thoracic shape and respiratory function. J Phys Ther Sci.

[7] Triangto K, Widjanantie SC, Nusdwinuringtyas N (2020). Biomechanical impacts of forward head posture on the respiratory function. IndoJPMR.

[8] Saxena A, Saraf A (2024). Forward head posture and its negative impact on respiratory function: a review study. JCHR.

[9] Hamasaki H (2020). Effects of diaphragmatic breathing on health: a narrative review. Medicines (Basel).

[10] Jahan I, Begum M, Akhter S (2021). Effects of alternate nostril breathing exercise on cardiorespiratory functions in healthy young adults. Ann Afr Med.

[11] Melo LC, Silva MA, Calles AC (2014). Obesity and lung function: a systematic review. Einstein (Sao Paulo).

[12] Miller MR, Hankinson J, Brusasco V (2005). Standardisation of spirometry. Eur Respir J.

[13] Dimitriadis Z, Podogyros G, Polyviou D, Tasopoulos I, Passa K (2015). The reliability of lateral photography for the assessment of the forward head posture through four different angle-based analysis methods in healthy individuals. Musculoskeletal Care.

[14] Sunitha G, Ravi BN (2013). Effect of deep breathing on respiratory parameters in healthy young individuals. J Evol Med Dent Sci.

[15] Woo SD, Kim TH, Lim JY (2016). The effects of breathing with mainly inspiration or expiration on pulmonary function and chest expansion. J Phys Ther Sci.

[16] Quanjer PH, Stanojevic S, Cole TJ (2012). Multi-ethnic reference values for spirometry for the 3-95-yr age range: the global lung function 2012 equations. Eur Respir J.

[17] Sheikhhoseini R, Shahrbanian S, Sayyadi P, O'Sullivan K (2018). Effectiveness of therapeutic exercise on forward head posture: a systematic review and meta-analysis. J Manipulative Physiol Ther.

[18] Ruivo RM, Pezarat-Correia P, Carita AI (2014). Cervical and shoulder postural assessment of adolescents between 15 and 17 years old and association with upper quadrant pain. Braz J Phys Ther.

[19] Lee DY, Nam CW, Sung YB, Kim K, Lee HY (2017). Changes in rounded shoulder posture and forward head posture according to exercise methods. J Phys Ther Sci.

